# Pharmacological Aspects and Biological Effects of Cannabigerol and Its Synthetic Derivatives

**DOI:** 10.1155/2022/3336516

**Published:** 2022-11-08

**Authors:** Fabrizio Calapai, Luigi Cardia, Emanuela Esposito, Ilaria Ammendolia, Cristina Mondello, Roberto Lo Giudice, Sebastiano Gangemi, Gioacchino Calapai, Carmen Mannucci

**Affiliations:** ^1^Department of Chemical, Biological, Pharmaceutical and Environmental Sciences, University of Messina, Messina 98168, Italy; ^2^Department of Human Pathology in Adulthood and Childhood “G. Barresi”, University Hospital “G. Martino” of Messina, Via Consolare Valeria 1, Messina 98123, Italy; ^3^Genetics and Pharmacogenetics Unit, Policlinico Universitario “G. Martino”, University of Messina, Messina 98125, Italy; ^4^Department of Biomedical and Dental Sciences and Morphofunctional Imaging, University of Messina, Messina 98125, Italy; ^5^Department of Clinical and Experimental Medicine, Unit and School of Allergy and Clinical Immunology, University of Messina, Messina 98125, Italy

## Abstract

Cannabigerol (CBG) is a cannabinoid from the plant *Cannabis sativa* that lacks psychotomimetic effects. Its precursor is the acidic form, cannabigerolic acid (CBGA), which is, in turn, a biosynthetic precursor of the compounds cannabidiol (CBD) and Δ9-tetrahydrocannabinol (THC). CBGA decarboxylation leads to the formation of neutral cannabinoid CBG, through a chemical reaction catalyzed by heat. On the basis of the growing interest in CBG and with the aim of highlighting scientific information on this phytocannabinoid, we focused the content of this article on its pharmacokinetic and pharmacodynamic characteristics and on its principal pharmacological effects. CBG is metabolized in the liver by the enzyme CYP2J2 to produce hydroxyl and di-oxygenated products. CBG is considered a partial agonist at the CB1 receptor (*R*) and CB2R, as well as a regulator of endocannabinoid signaling. Potential pharmacological targets for CBG include transient receptor potential (TRP) channels, cyclooxygenase (COX-1 and COX-2) enzymes, cannabinoid, 5-HT1A, and alpha-2 receptors. Pre-clinical findings show that CBG reduces intraocular pressure, possesses antioxidant, anti-inflammatory, and anti-tumoral activities, and has anti-anxiety, neuroprotective, dermatological, and appetite-stimulating effects. Several findings suggest that research on CBG deserves to be deepened, as it could be used, alone or in association, for novel therapeutic approaches for several disorders.

## 1. Introduction

**Figure 1:**
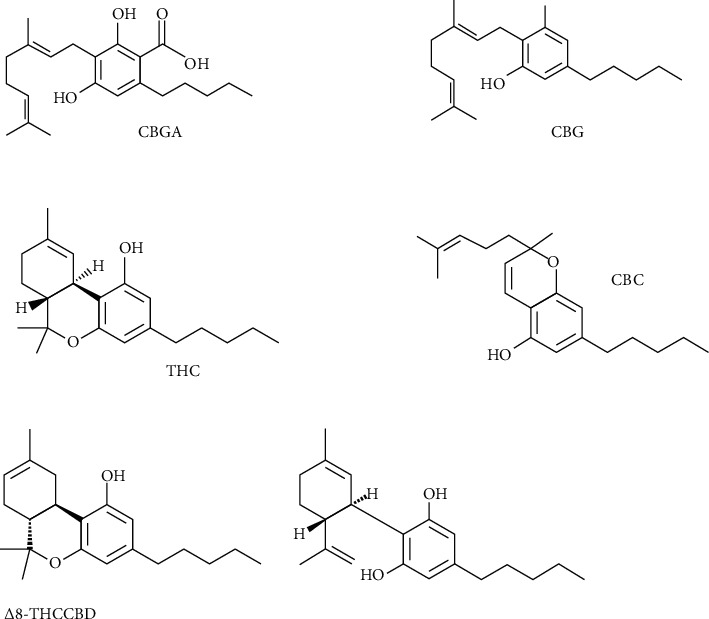
Major phytocannabinoids.

The increased therapeutic potential of *Cannabis sativa* L. (fam. Cannabaceae; *C. sativa*) and the pharmacology of its chemical constituents need a more in-depth understanding in terms of its other constituents, rather than the more known phytocannabinoids Δ9-tetrahydrocannabinol (THC) and cannabidiol (CBD) (Figure 1). After the isolation of THC, the main psychoactive constituent of *C. sativa* [1, 2], over 100 phytocannabinoids have been found in this plant, one of these being cannabigerol (CBG) [3].

A few years after the identification of THC (Figure 1), *in vivo* assays showed that the phytocannabinoid CBG is not psychotomimetic like the more well-known phytocannabinoid THC [4, 5]. However, it has been neglected and shadowed by THC for years because of its lower concentration and, paradoxically, probably for its lack of psychotomimetic activity. CBG is the terpenophenolic phytocannabinoid precursor, in the plant *C. sativa*, of both THC and CBD [6]. CBG is not present only in this plant; beyond its discovery in *C. sativa*, it has also been found in the phytochemical profile of an extract from *Helichrysum umbraculigerum*, considered to be the most abundant source of CBG [7] (Figure 1).

In the plant, the cannabinoids THC and CBD are synthesized in an acidic (carboxylated) form, namely, cannabigerolic acid (CBGA) (Figure 1). CBGA undergoes a decarboxylation process depending on different factors, in particular, the speed of this process is related to the rise in temperature (Figure 1). Decarboxylation causing CBG formation is due to a simple chemical reaction catalyzed by heat, a conversion taking place at room temperature, but in a much slower way [8]. This intermediate cannabinoid is not present at significant concentrations in *Cannabis*, commonly occurring as a minor compound in terms of proportions [9, 10]. As happens for THC and CBD, the plant primarily synthesizes CBGA; this acidic form of the cannabinoid is unstable from the thermic point of view and can be decarboxylated when exposed to light or heat via smoking, baking, or refluxing [11]. In this article, we highlight the current scientific information on CBG, focusing on its pharmacokinetic and pharmacodynamic characteristics and on its principal pharmacological effects, and on pharmacological effects of CBG-derived compounds.

## 2. Methods

Bibliographic research was carried out independently by two researchers using major scientific databases (PubMed, Scopus, and Google Scholar) using the keyword “cannabigerol” is discussed in the present article. The investigators used the keyword “cannabigerol.” Articles written in the English language and published in peer-reviewed scientific journals describing the pharmacological aspects of cannabigerol published between January 1964 and December 2021 were collected and discussed.

### 2.1. Pharmacokinetics of Cannabigerol

**Figure 2:**
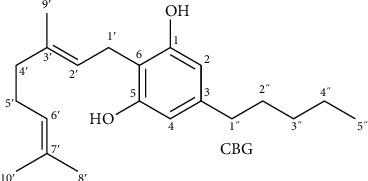
CBG numbering system.

The pharmacokinetics of CBG has not been still sufficiently characterized in humans. However, there are known methods to quantify CBG presence in the blood [12], such as the liquid chromatography (LC) method, which seems to be more suitable than the gas chromatography (GC) method to measure cannabinoids, because it allows contemporary analysis of acidic and neutral forms. The reason is that acid forms keep their original form during LC analytical conditions, while they turn into neutral form due to the GC temperature conditions [13] (Figure 2).

CBG has been quantified by LC-tandem mass spectrometry in the oral fluid of frequent and occasional cannabis smokers. The maximum concentration (*C*_max_), time to *C*_max_ (*T*_max_), and time of last detection (Tlast) have been calculated, showing that CBG Tmax occurs at approximately 0.17 h after cannabis intake, with a successive quick decrease. It can be detected either in frequent or occasional smokers. In frequent smokers, CBG Cmax is greater if inhaled than after the oral intake of cannabis; meanwhile, no difference has been reported between smoked and vaporized cannabis. In frequent smokers, CBG Tlast occurs significantly later after smoking and vaporizing compared to oral administration, thus suggesting that CBG remains longer in the blood circulation after inhalation compared to the oral route (not clear). Generally, CBG cannot be detected beyond 26 hours after any way of administration. In summary, CBG exhibits higher concentrations in the oral fluid of frequent smokers after inhalation than after the oral intake of cannabis [14].

CBG, in the acidic form, is the forerunner of cannabinoids following condensation of geranyl phosphate and olivetolic acid in *C. sativa*. Its identification has been allowed by chemical analysis of urine samples of *Cannabis* consumers. CBG entry in the body occurs with *Cannabis* smoking and its conjugated form is eliminated with urine, as happens for other neutral cannabinoids. Previous chromatographic analysis of hydrolyzed and trimethylsilylated urine samples found a chemical substance in *Cannabis* consumers' urine extracts, with fragment ions at *m*/*z* 425, 465, and 479 at a retention time of 14.19 min, which is presumed to be 4″-hydroxy-CBG or 5″-hydroxy-CBG. This was not found in non-hydrolyzed urine samples, suggesting that CBG is also eliminated in the glucuronidated form [15]. The detection rates of CBG in frequent smokers have been shown to be 100% and 90.9% after smoking and vaporization, respectively. Meanwhile, in occasional smokers, the CBG detection rates were 77.8% and 66.7% after smoking and vaporization, respectively. Following detection in frequent smokers (*p*=0.015), Tlast was significantly later than in infrequent smokers in a way not dependent on the inhalation method. CBG detection in constant smokers showed considerably greater *C*_max_ (*p*=0.046) and later Tlast (*p*=0.026) values following smoking in comparison to vaporization, partially due to ineffective CBG evaporation during vaporization. Following cannabis intake, CBG is traceable in the urine where it could be used as an index of current use after inhalation [16].

CBG is considered a potent competitive inhibitor of anandamide (AEA). As occurs for other chemicals, the flexible nature of CBG allows it to occupy most of the active site volume, which could explain its competitiveness versus AEA. CBG competitively inhibits AEA metabolism with a Ki of 10.8 ± 1.4 *μ*M, whereas THC, Δ8-THC, CBD, cannabinol (CBN), and cannabichromene (CBC) behave as noncompetitive AEA inhibitors. Among these substances, THC is the most powerful inhibitor of the AEA metabolism and causes a reduction of the CYP2J2-mediated AEA metabolic transformation to 20% of the uninhibited activity [17].

CBG, as occurs for Δ9-THC, Δ8-THC, CBC, CBD, and CBN (Figure 1), is hydroxylated by the isoenzyme CYP2J2. Metabolite formation has been investigated in mice, rats, cats, guinea pigs, hamsters and gerbils, and rabbits. It has been observed that each species is responsible for the formation of similar substances, even if ratios of single metabolites are significantly different [18]. The research identified twelve products of metabolism, among these ones the most important being monohydroxy compounds showing the hydroxyl group at C-8′, C-9, C-4′, or at positions of the pentyl chain. Metabolites are similar to products of metabolism detectable by investigating other cannabinoids and hydroxylation and epoxidation are the most significant *in vitro* pathways. The principal metabolic pathway in most species is generally allylic hydroxylation of the trans-terminal methyl group (C-8′) [19]. A previous study of CBG metabolism in rats, guinea pigs, rabbits, and hamsters showed that this cannabinoid receives allylic hydroxylation at the terminal's double-bond of the C-10 chain, producing the principal metabolites in all species except for mice, where occurs the formation of an epoxide at the terminal's double-bond. In guinea pigs hydroxylation of the pentyl side-chain, particularly at C-3″, was prominent. In other species, 4″-hydroxylation was the undertaken major biotransformation route [20].

The pharmacokinetic profile of CBG after oral and intraperitoneal acute single-dose administration has been investigated in the blood and brains of rats and mice. The maximal drug concentration in tissue (*C*_max_), the time to get to the maximal tissue levels (*T*_max_), apparent elimination (depending also on the rate of absorption and distribution), half-life (2–6 h after oral administration; per os; 4–24 h after intraperitoneal administration; i.p.), area under the curve (AUC), and brain/plasma ratios founded on the AUC (0–6 h) were measured in the blood and brain specimens at six-time points after i.p. and oral CBG. Rapid absorption was observed both for i.p. and oral CBG administration in the plasma and brain of mice. CBG i.p. injections produced significantly higher plasma and brain concentrations in comparison to oral intake and *T*_max_ was detected at 30 min in the plasma and 120 min in the brains, while the AUCs were approximately 100-fold greater in comparison to oral way of administration. However, after i.p. injections, the concentrations in both tissues did not show any considerable differences; after oral administration, the concentrations were extremely low and the brain/plasma ratios were 0.77 and 0.15 for the oral and i.p. way of administration, respectively. Moreover, in rats, CBG was quickly absorbed following i.p. and oral administration in both the plasma and the brain, with *T*_max_ between 30 and 120 min, but i.p. administration provided better exposure, leading to a lower concentration detected following oral intake and stable brain exposure 2–4 h after treatment, with i.p. versus per os brain/plasma ratios overlapping [21].

AEA is converted by different CYP epoxygenases, including CYP2J2, into 14, 15-epoxyeicosatrienoic acid-ethanolamides (EET-EAs) [22]. It has been observed that phytocannabinoids can reduce the metabolism of endogenous substrates such as AEA by inhibiting CYP2J2 [23].

Summarizing the pharmacokinetic research on CBG, it is possible to assert that after inhalation of cannabis, CBG is present in the plasma after a few minutes and reaches *T*_max_ at approximately 0.17 h, followed by a rapid reduction in concentration. It is absorbed rapidly after i.p. and per os administration. After a single oral administration, its half-life is 2–6 h, and it has also been found in the brain. CBG undergoes a metabolictransformation in the liver, and the major metabolic route is allylic hydroxylation. CBG is metabolized by the enzyme CYP2J2 to produce new hydroxyl and de-oxygenated products. The major metabolites are monohydroxy compounds. CBG is excreted with urine in the conjugated form, as occurs for other cannabinoids.

### 2.2. Pharmacodynamics of Cannabigerol

Cannabinoid receptors CB1R and CB2R are both associated with inhibitory Gi/o proteins to adenylyl cyclase, and with the mitogen-activating protein (MAP) kinase in an activating way [24]. After receptor activation by phytocannabinoids, the cyclic adenosine monophosphate (cAMP) level is reduced by inhibitory adenylate cyclase and stimulatory MAP kinase. This causes the shortening of the presynaptic action potential, thus inhibiting Ca2+ entry and activating the enzymes phospholipases A and C, thus leading to a decrease in excitatory and inhibitory transmitter discharge [25].

CBG binds itself to CB1R and CB2R [26] and is considered a partial agonist at these receptors (*K*i values ranging from 300–500 nM) [27]. Other authors have demonstrated that CBG is a poor ligand, binding weakly without activation of CB1R and CB2R, while a sesquicannabigerol, a farnesyl prenylogue of cannabigerol, has been reported to have a higher affinity at CB2R [28]. Other experiments have shown the binding of CBG to CB1R (*K*i = 381 nM) in mouse brain membranes and CB2R (*Ki* = 2.6 *μ*M) in CHO cells [29]. It has been shown that CBG binding to CB2R produces a less efficacious CB2R-mediated inhibition of cyclic AMP than THC [27].

CBG shares a similar mechanism of action with the cannabinoid CBD, since it binds itself weakly to CB1R and CB2R and, in addition to blocking CB1R, antagonizes 5-HT1A receptor, activates alpha (2) adrenoceptors [30, 31], and is a modulator of endocannabinoid signaling [30]. CBG acts as a strong *α*2-adrenoceptor agonist (EC_50_ = 0.2 nM), while it is only a moderate 5-HT1AR competitive antagonist, and at high concentrations (10 *μ*M), it antagonizes [35S] guanosine 5′-O-[gamma-thio]triphosphate (GTP*γ*S) binding in mouse brain membranes stimulated with AEA or CP-55940 [29, 30].

It has also been demonstrated that CBG causes inhibition of the reuptake of AEA and acts as an agonist in the TRP channels TRP ankyrin (A) 1, TRP vanilloid (V) 1, and TRPV2, while is an antagonist of the TRP subfamily melastatin (M) 8. Previous findings have shown that CBG can act as an agonist/desensitizer of transient receptor potential (TRP) ankyrin (A) 1 (EC_50_ = 700 nM), an agonist of TRP (vanilloid) V1 (EC_50_ = 1.3 *μ*M), an agonist of TRPV2 (EC_50_ = 1.7 *μ*M), an antagonist of TRPmelastatin (M) 8 channels (IC_50_ = 160 nM), and an inhibitor of AEA cell uptake (*Ki* = 11.3 *μ*M) [32]. CBG may also modulate the activity of TRP of ankyrin type-1 channels; however, the EC_50_ values lie in the micromolar range [33]. CBG is an antagonist of TRPM8, useable for possible employment in the treatment of prostate cancer, bladder overactivity, and bladder pain [34, 35].

The peroxisome proliferator-activated receptor (PPAR) *γ* has also been indicated as a potential pharmacological target of CBG (*Ki* = 11.7 *μ*M), and concentrations of CBG between 10 and 25 *μ*M produce enhancement of PPAR*γ* transcriptional activity [36, 37]. CBG also blocks the voltage-gated sodium channels Nav 1.1, 1.2, and 1.5 and can reduce the psychotropic effect of THC [38].

Both cannabinoids and endocannabinoids possess anti-inflammatory activity through a not sufficiently clarified mechanism of action. It has been suggested that anti-inflammatory activity is a key factor in the development of colon cancer. COX-mediated anti-inflammatory activity of CBG has been studied by using a concentration of 2.5 · 10–5 M, while CBGA has been tested at a concentration of 6.25 · 10–5 M. Both CBG and CBGA showed inhibition of the enzyme COX-1 more than a 30%. The same percentage (30%) of inhibition was shown by CBG and CBGA on COX-2 enzyme activity. Anyway, CBG and CBGA inhibition of prostaglandin production resulted to be law being less than 10%. In conclusion, experiments have shown that CBG inhibits COX enzymes in a higher concentration range, compared to conventional anti-inflammatory compounds [39].

## 3. Biological Effects

### 3.1. Anti-Inflammatory and Antioxidant Activities

The endocannabinoid system modulates homeostatic processes, including gastrointestinal motility, hunger, pain perception, and immunity [40]. Anti-inflammatory and anti-oxidant activities were investigated mainly through pre-clinical research. In laboratory animals investigation, cannabinoids have been shown to have anti-inflammatory effects, and a subgroup analysis suggested that in experimental colitis, CBG caused the largest reduction in myeloperoxidase activity and in the effect size (SMD −6.20; 95% CI, −9.90 to −2.50), followed in producing these effects by the synthetic CB1 agonist arachidonyl-2-chloroethylamide (ACEA) [41].

Inflammatory bowel disease (IBD) includes chronic relapsing inflammatory disorders Crohn's disease (CD) and ulcerative colitis. The effects of CBG were studied in experimental colitis induced in rodents by intracolonic dinitrobenzene sulphonic acid (DNBS). CBG (1–30 mg/kg) i.p. administration before or following induction of experimental colitis significantly improved the consequences of DNBS administration on colon weight/length ratio, an index used for the evaluation of tissue edema caused by inflammation [42]. The effects of CBG on intestinal inflammation have also been studied by assessing myeloperoxidase activity, histological evaluation and immunohistochemistry, measurement of interleukin (IL)-1*β*, IL-10 and interferon (IFN)-*γ* levels by ELISA, inducible nitric oxide synthase (iNOS), and COX-2, CuZn-superoxide dismutase (SOD) activity. Nitric oxide production and oxidative stress have been evaluated by measuring the effects of CBG on murine macrophages and intestinal epithelial cells, respectively. The results showed that CBG reduces the weight/length ratio of the colon, myeloperoxidase activity, and iNOS expression, and these results were associated with increased SOD activity and normalization of the IL-1*β*, IL-10, and IFN-*γ* modifications caused by DNBS administration. In macrophages, CBG caused reduction of nitric oxide production and iNOS protein (but not mRNA) expression. Interestingly, the CB1R antagonist rimonabant did not modify CBG effects on nitric oxide formation, while the CB2R antagonist SR144528 enhanced the inhibition of CBG on nitric oxide production. According to the authors' conclusions, these experiments show that CBG treatment is able to reduce experimental colitis through a reduction in nitric oxide formation in macrophages via CB2R activation and the production of a diminution of reactive oxygen species (ROS) in intestinal epithelial cells [43].

Other authors have confirmed that cannabinoids inhibit oxidative and nitrosative stress through the modulation of iNOS expression and reduction of ROS [44, 45], and that CBG exerts antioxidant activity comparable to that of vitamin E [46]. The antioxidant activity counteracting hydrogen peroxide (H_2_O_2_)-induced oxidative stress in murine RAW264.7 macrophages incubated with CBG has been investigated. It was found that 10 *μ*M of CBG inhibited oxidative stress after stimulation of CB2R, since pre-treatment with the CB2R antagonist AM630 antagonized the protective effects of CBG occurring in H_2_O_2_-stimulated macrophages, while the involvement of CB1R seems to be excluded. CBG antioxidant activity implies the downregulation of oxidants signals iNOS, nitrotyrosine, and PPAR-1 by preventing I*κ*B-*α* phosphorylation and the translocation of nuclear factor-*κ*B (NF-*κ*B), as well as the regulation of the MAP kinase pathway. Contextually, it has been found that CBG increases cellular antioxidant defense by modulating SOD-1 expression, thus inhibiting cell death [47]. This indicates that CBG could be useful as a new approach in the care of oxidative stress-related disorders. CBG may effectively work as a free radical scavenger to enhance cellular antioxidant activity through the modulation of pathways such as MAPK kinase and NF-*κ*B translocation and to counteract cell death.

In the view that inflammation and oxidative stress also have a key role in neurodegeneration, it has been investigated whether CBG may have neuroprotective effects counteracting inflammation and oxidative stress, thus exerting protection against neuronal loss. The capability of CBG to defend neuroblastoma spinal cord (NSC)-34 motor neurons from the toxicity induced in lipopolysaccharide (LPS)-stimulated RAW264.7 macrophages has been evaluated.

Through an *in vitro* MTT [3-(4, 5-dimethylthiazol-2-yl)-2, 5-diphenyl-2H-tetrazolium bromide)] assay (to measure cellular metabolic activity as an indicator of cell viability, proliferation, and cytotoxicity), it was observed that CBG (1–20 *μ*M) reduced the loss of cell viability induced by LPS-stimulated macrophages in NSC-34 cells. In these experiments, CBG pre-treatment reduced apoptosis, as revealed by a decrease in caspase 3 activation and nuclear-encoded protein Bax expression, while the anti-apoptotic protein Bcl-2 was more elevated. Moreover, CBG pre-treatment reduced inflammation, as demonstrated by the reduction in the IL-1*β*, tumor necrosis factor-*α* (TNF)-*α*, IFN-Υ, and PPAR protein levels evaluated by immunocytochemistry, but also oxidative stress in NSC-34 cells treated with the medium of LPS-stimulated RAW264.7. These experiments suggest that CBG may be a potential treatment to be used against neuroinflammation and oxidative stress [48].

### 3.2. Antitumoral Activity

Effects of CBG were studied on several tumor cell lines. The antitumoral activity of CBG against human oral epithelioid carcinoma cells has been investigated. In these *in vitro* experiments, CBG showed an antitumoral property against skin melanoma cells with significant activity (IC_50_ = 31.30 *μ*M) in an *in vitro* MTT (3-(4, 5-Dimelhyllhiazol-2-yl)-2, S-diphenyl-2 H-tetra-zoliumbromide) microculture assay [49]. The same authors observed that CBG inhibited the growth of mouth epidermal carcinoma cell lines, producing an IC_50_ of 31 *μ*M in an MMT assay and 77 *μ*M in a sulforhodamine B protein assay. In these experiments, CBG showed the least cytotoxic effect on murine NIH 3T3 cells and was more potent than 5-fluorouracil [50]. CBG was found to be effective against breast cancer in the human MDA-MB-231 cell line [51] and to antagonize proliferation of the hyper-proliferating human keratinocyte cell line [52]. Furthermore, as observed above, CBG is able to reduce experimental intestinal inflammation, which might be important in light of the risk of developing colorectal cancer (CRC), which is considerably more elevated in ulcerative colitis patients [53]. From this point of view, the effects of CBG on CRC cell growth and its potential preventive activity in an azoxymethane model of colon cancer, as well as its potential therapeutic effect in a xenograft model of colon cancer, were evaluated. In this experimental model, it was shown that CBG promoted apoptosis, stimulated ROS production, upregulated the mRNA of transcription factor CCAAT-enhancer-binding protein homologous protein (CHOP), and slowed down the cell growth in CRC cells. The effects of CBG on cell growth were not dependent on TRPA1, TRPV1, and TRPV2 channel activation, they were additionally enhanced by the CB2R antagonist SR144528, and were produced also by other TRPM8 channel blockers but not by the 5-HT1A antagonist rimonabant. CBG activity on cellular growth and CHOP mRNA expression was diminished in the TRPM8 cellular line. CBG antagonized the development of xenograft cancers and colon tumors induced by chemicals. In the same experimental model, CBG hampered tumor progression and inhibited the progress of CRC cells [54]. These *in vivo* experiments show that CBG antagonizes the growth of CRC cells principally by stimulating apoptosis and hindering the development of colon carcinogenesis. Moreover, authors have suggested that TRPM8 is also at least partially involved in the antitumoral activity of CBG. The suppressor activity of CBG of the growth on tumor cell lines is accompanied by ROS hyperproduction and seems to be partially mediated by TRPM8. The apparent safety and experimental results of CBG indicate that it could be promising for preventing and treating CRC.

Glioblastoma is aggressive brain cancer, showing increasing incidence. It has been found that CBG, as occurs with THC, slows the progression of this tumor and inhibits the invasion of glioblastoma cells. Furthermore, CBG is effective in reducing therapy-resistant glioblastoma stem cells [55]. These findings indicate that CBG, also showing analgesic, anti-nausea, and appetite-stimulating effects, could represent a new adjuvant therapy for glioblastoma.

### 3.3. Neuroprotective Effects

CBG as a cannabis-derived chemical not causing psychotomimetic effects is interesting as a potential new drug for central nervous system (CNS) pathologies.

Neuroprotective effects of CBG and CBD have been compared in experiments simulating oxidative stress and neurotoxicity as they occur in neurological pathologies in rats. CBG and CBD exert antioxidant activity in astrocytes exposed to hydrogen peroxide and restored the content of serotonin in the cortex [56].

The effects of CBG have also been studied on human brain microvascular endothelial cells (HBMECs), pericytes, and astrocytes forming the blood-brain barrier (BBB) under ischemic conditions. Through this experimental model, it has been shown that 10 *μ*M of CBG reduced IL-6, lactate dehydrogenase, and DNA damage protein levels in astrocytes [57].

The protective effects of CBG have been investigated in two *in vivo* models of Huntington's disease (HD) induced by 3-nitropropionate (3NP). CBG administered through daily four i.p. injections (10 mg/kg) in mice improved motor deficits, preserved striatal neurons, attenuated microgliosis, and reduced inflammatory markers induced by 3NP against 3NP toxicity, and ameliorated antioxidant activity. CBG also produced significant recovery in the deteriorated rotarod performance. Furthermore, by using HD array analysis, it was evident that CBG turned to normal a series of genes related to this pathology [58].

**Figure 3:**
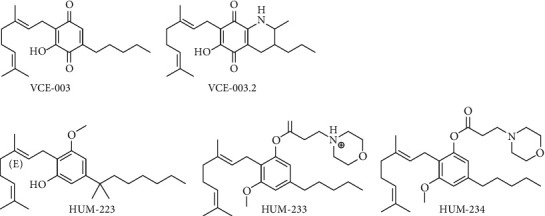
CBG pleiotropic mechanism of action.

The synthetic analog cannabigerol quinone VCE-003 (Figure 3), obtained by oxidation modification in the resorcinol moiety of the compound, has been identified as an anti-inflammatory substance. VCE-003 reduced the effects of excitotoxicity on neuronal cells, activated PPAR*γ* transcriptional activity, and antagonized the release of pro-inflammatory substances in an experimental model characterized by the stimulation of microglial cells with lipopolysaccharide (LPS). In the same series of experiments, the authors found that VCE-003 improved the symptoms of a model of multiple sclerosis (MS) represented by Theiler's murine encephalomyelitis virus (TMEV) infection [59].

Multiple sclerosis (MS) is a chronic inflammatory demyelinating CNS disease, considered a predominantly T cell-mediated autoimmune disease and neuroinflammation is a fundamental component in the pathophysiology of this disease [60]. It has been demonstrated that VCE-003, reduces neuroinflammation and motor deficiency in viral T cells and macrophages, and its effectiveness in an autoimmune model of MS has been investigated. Proliferation, cell cycle, and expression of activation markers were assessed by fluorescence-activated cell sorting analysis in human primary T cells, and cytokine and chemokine formation was quantified. In the same study, transcription was investigated by using Jurkat and RAW264.7 cells with the aim of evaluating VCE-003 activity on IL-17-induced macrophage polarization to activate the M1 phenotype. Experimental autoimmune encephalomyelitis (EAE) was induced by myelin oligodendrocyte glycoprotein (MOG35–55) immunization and spinal cord harm was established with immunohistochemistry. The results showed that the postimmunization administration of i.p. VCE-003 (5 mg/kg) for 21 consecutive days inhibited proliferative effects stimulated by CD3/CD28, cell cycle progression, and induction of IL-2Ra and ICAM-1 in human T cells. VCE-003 also inhibited the formation of Th1/Th17 cytokines and chemokines in murine T cells. This effect was associated with a reduction of transcription of IL-2, IL-17, and TNF-*α* promoters when induced by CD3/CD28. Moreover, VCE-003 and the CB2 agonist JWH-133, attenuated the pro-inflammatory polarization of macrophages caused by IL-17. Another effect of VCE-003 was the prevention of iNOS expression stimulated by LPS. VCE-003 ameliorated the neurological profile and harshness of EAE in mice by stimulation of CB2 and PPARc receptors. Finally, in these experiments, attenuation of cell infiltrates, associated with reduction of microglial activation, structural conservation of myelin, and protection of axons, were observed. Thus, indicating the potential role of VCE-003 as a therapeutic compound to use in immune diseases characterized by inflammation [61].

The analog VCE-003.2 (Figure 3), another CBG quinone derivative, acting through PPAR*γ* (a nuclear receptor involved in the lipidic metabolism and glucose homeostasis), has been studied for its potential neuroprotection. It has been shown that it favors the pro-survival of progenitor cells all along with neuronal differentiation, and this effect was interfered with by PPAR*γ* antagonists. Moreover, VCE-003.2 given i.p. at the dose of 10–20 mg/kg reduced cell death caused by quinolinic acid (QA), activation of caspase-3 activation, and accumulated mutant huntingtin aggregates in cells originating from the striatum. In the *in vivo* Huntington's-like disease models characterized by striatal neurodegeneration induced by QA and 3NP, VCE-003.2 prevented medium spiny DARPP32+ neuronal loss, ameliorated motor deficits, reactive astrogliosis, and microglial activation. In the 3NP model, VCE-003.2 antagonized the formation of proinflammatory markers and improved brain antioxidant activity. Taken together, the results of these experiments bring to consider the compound VCE-003.2 as a potential candidate for the therapy of HD and other neurodegenerative pathologies characterized by the presence of inflammatory components [62].

Neuroprotection of VCE-003.2 in amyotrophic lateral sclerosis (ALS) was investigated by using SOD1G93A mutant mice. VCE-003.2 (10 mg/kg) was administered i.p. from 60 days up to 18 weeks when the disease is in an advanced stage. Treatment with VCE-003.2 decreased neurological abnormalities, protected spinal cholinergic motor neurons, and attenuated astrogliosis. Elevation in IL-1*β* and glial glutamate transporters, as well as the LPS-induced release of TNF-*α* and IL-1*β* in cultured astrocytes of SOD1G93A transgenic newborns, were reduced, probably through PPAR-*γ* stimulation. The results showed the neuroprotective effects of VCE-003.2 in SOD1G93A transgenic mice, indicating PPAR-*γ* as an additional therapeutic target suitable for the utilization of cannabinoids in ALS [63].

The effects of VCE-003.2 were investigated *in vivo* in mice in which unilateral intrastriatal lesions were caused by the injection of LPS to reproduce a model of Parkinson's disease (PD), as well as in an *in vitro* model (LPS-exposed microglial BV2 cells and immortalized rat embryonic striatal M-213 cells treated with media obtained from LPS-exposed BV2 cells). I.p. administration of VCE-003.2 (10 mg/kg), alone or in association with the PPAR*γ* antagonist T0070907 (5 mg/kg) given for 21 days beginning 16 h after LPS lesion, decreased the loss of nigrostriatal neurons and the intense microgliosis in the *Substantia nigra*, measured by Iba-1/Cd68 immunostaining. Mediators of inflammation TNF-*α*, IL-1*β*, and iNOS in the striatum showed marked elevation by the LPS lesion and were significantly reduced by VCE-003.2 via activation of PPAR*γ*. Moreover, the *in vitro* approach confirmed the anti-inflammatory activity of VCE-003.2, showing that in LPS-exposed BV2 cells it reduced the synthesis and release of TNF-*α* and IL-1*β*, and the induction of iNOS and COX-2 [64].

More recently, VCE-003.2 has been investigated in mice in another model of PD, in this case, induced with the neurotoxin 6-hydroxydopamine (6-OHDA), comparing its effects with two other CBG-related derivatives, cannabigerolic acid quinone (CBGA-Q) and its sodium salt (CBGA-Q-Salt). These compounds, as well as VCE-003.2, act at PPAR-*γ* receptor, but are not ligands for CB1R and CB2R. VCE-003.2 was cytoprotective in the SH-SY5Y cell line exposed to 6-OHDA at a concentration of 20 *μ*M, an effect not changed by the PPAR-*γ* receptor antagonist GW9662. Cytoprotection was also observed with CBGA-Q and CBGA-Q-Salt; however, this effect was abolished by GW9662. *In vivo* experiments showed that the oral administration of VCE-003.2 (20 mg/kg) protected nigral neurons against 6-OHDA-induced damage. This neuroprotection was accompanied by motor deficiencies caused by 6-OHDA. Similar protection was observed with CBGA-Q, given orally (20 mg/kg) or intraperitoneally (10 mg/kg, i.p.), but to a lower extent, while CBGA-Q-Salt (10 mg/kg) was weakly active. The authors' interpretation emphasized the role of PPAR-*γ* receptors involvement in these effects [65].

The protective effects of VCE-003.2 have also been studied in striatal neurodegeneration by using *in vivo* adeno-associated viral expression of mutant huntingtin and *in vitro* mouse embryonic stem cell differentiation. The effects of VCE-003.2 on embryonic stem-neuronal differentiation were investigated. The R1 line of mouse ES cells was treated with VCE-003.2 during neural differentiation for 21 days and transcription factor CTIP2-positive striatal medium spiny neurons were examined. Immunofluorescence showed that VCE-003 enhanced the quantitative of CTIP2-positive cells as well as doublecortin immunoreactivity and favored differentiation. In *in vivo* experiments, daily orally administered VCE-003.2 (10 mg/kg) protected striatal neurons from injury caused by mutant huntingtin, reduced neuroinflammation, and ameliorated motor deficit. Other effects of VCE-003.2 were the promotion of mobilization of subventricular zone progenitor and of migration of neuroblasts toward the damaged area, and increase in neurogenesis. VCE-003.2 also increased neuroblasts and striatal-like neurogenesis. In summary, VCE-003.2 improved subventricular zone-derived neurogenesis against neurodegeneration caused by mutant huntingtin, and was shown to be neuroprotective upon oral administration [66].

### 3.4. Anti-Anxiety Effects

Endocannabinoids have been shown to modulate feeding and emotional behaviors [67, 68]. CBG acts as a 5-HT1A antagonist as demonstrated in *in vitro* [30] and *in vivo* experiments [69]. For this reason, it has been proposed that it can be responsible for the anti-anxiety effects of cannabis [70]. However, the notion that CBG behaves as a 5-HT1A antagonist conflicts its putative anxiolytic effects because an agonist—or partial agonist—would be more likely to produce anxiolytic effects. These aspects were investigated by comparing the effects of acute and chronic (14 days) i.p. CBG (2.5 mg/kg) with the effects of chronic i.p. administration of THC (2.5 mg/kg) and CBD (2.5 mg/kg) on the time consumed in an open, lit box in a light–dark (LD) immersion model of anxiety-like behavior and saccharin hedonic reactions in a taste reactivity test of palatability processing in rats. The results showed that THC caused acutely an anxiogenic-like behavior in the LD immersion test, not enhanced by chronic administration. THC increased only transiently on the first day of administration the saccharin palatability in the taste reactivity test. Both CBD and CBG did not change the anxiety-like response but CBG produced a light increase of saccharin palatability only on the first day of administration. These results were unable to corroborate the anti-anxiety effects of CBG in rats because they did not show the anxiolytic effect of CBG [71]. Contrary to these results, in other experiments, it has been observed that i.p. CBG administration at a dose of 10 mg/kg in mice enhanced the period of time consumed in the central quadrant of the open field test, thus suggesting potential anxiolytic effects. In the same group of experiments, i.p. CBG administration at a dose of 3 mg/kg also produced a light anti-nociceptive effect [72].

### 3.5. Anti-Nausea Effects

CBG seems to influence the anti-emetic properties of THC. Antagonism of CBG toward CB1R and 5-HT1A receptors [30] abolishes the anti-emetic activity of low-dose of CBD, probably because it is due to action at the 5-HT1A receptor level [64]. The potential of CBG to antagonize the anti-emetic effect of the 8-OH-DPAT antagonist of the 5-HT1A receptor [73], has also been assessed. The emetic effects properties of higher doses of CBD and CBG may have involved in the production of severe nausea and vomiting when cannabinoid hyperemesis syndrome occurs [74]. Experiments have been conducted to study and evaluate possible CBG effects either in regulating nausea in rats or vomiting in *Suncus murinus*. Two experiments were carried out; in experiment 1, rats received i.p. CBG (0.0, 1, 5, and 10 mg/kg) 15 min prior to receive i.p. vehicle or CBD (5 mg/kg; experiment 1a) or i.p. vehicle or 8-OH-DPAT (0.01 mg/kg; experiment 1b). After 30 min, all rats were i.p. treated with the association of 0.1% saccharin solution and LiCl (20 mL/kg of 0.15 M). Seventy-two hours later, animals were subjected to a saccharin taste reactivity to observe the effects on the establishment of conditioned gaping reactions (an experimental model of nausea) and conditioned saccharin avoidance. In experiment 2, *S. murinus* were i.p. treated with CBG (5 mg/kg) or vehicle 15 min before CBD (5 mg/kg) or vehicle, and 30 min later i.p. treated with LiCl (60 mL/kg of 0.15 M), to evaluate the number of vomiting episodes. CBD blocked conditioned gaping in rats and vomiting in shrews, and these effects were antagonized by pretreatment with all doses of CBG. CBG also antagonized the anti-nausea effects of 8-OH-DPAT. These results indicate that injection of moderate doses of CBG and CBD may be opposite to each other at the 5-HT1A receptor level with interference in the modulation of nausea and vomiting. These findings suggest that CBG could be used for its potential interactions with CBD due to its opposite action at the 5-HT1A receptor in nausea and vomiting [69].

### 3.6. Reduction in Intraocular Pressure (IOP)

A reduction of IOP in humans following smoked cannabis was noted in 1971 [75]. Successively, findings carried out in different species have shown that THC has an ocular hypotensive effect [76]. Cannabinol (CBN) or CBG have been studied in cats by topically administering them doses of 250, 500, and 1000 picograms as a single drop or through via osmotic minipumps (20 *μ*g/h) for nine days. CBN single dose had a moderate effect on IOP, but it produced a significant reduction if chronically administered. Similarly, CBG reduced IOP, but the size of the effect when it was chronically administered was greater. Furthermore, CBN but not CBG caused side effects such as conjunctival erythema and hyperemia. Following i.p. administration of CBN (20, 40, or 80 mg/kg) to rats, 8–13 Hz polyspike discharges were observed in the electrocorticogram during vigilance and sleep rapid eye movement episodes. Interestingly, systemically injected CBG (10, 30, and 100 mg/kg) lacked this effect. The above-cited results suggest that chronic administration of these cannabinoids reduces ocular tension. However, as happens for cannabis and THC, CBN can cause both ocular toxicity and neurotoxicity. As CBG did not produce these toxicities, it is evident that the ocular hypotensive effect obtained with this cannabinoid can be distinguished from both the adverse central and ocular effects associated with cannabis [77]. When CBG was chronically unilaterally delivered for 9 consecutive days to the eyes of cats through osmotic minipumps releasing it at a rate of 1 *μ*L/h, it produced a fall in IOP similarly to THC (4–7 mm Hg) applied in the same way. The same group of researchers observed that in rats, following peripheral administration of THC, but not CBG, polyspike discharge became visible in the cortical electroencephalogram during the wake. Then, polyspikes were detectable within rapid eye movement sleep periods. Results also showed that both cannabinoids caused a two-to three-fold increase in aqueous outflow [78]. As the greatest proportion of the drugs used for the therapy of glaucoma suppresses aqueous release, CBG could be a promising anti-glaucoma substance to be used with drugs acting by enhancing aqueous outflow instead by increasing of ocular fluid drainage.

IOP-lowering activity was studied in rats using a synthetic CBG analog, cannabigerol-dimethyl heptyl (CBG-DMH), compared to the nonselective CB1R and CB2R agonist, WIN55, 212-2. CBG-DMH i.p. administration reduced IOP at doses of 2.5 mg/kg or given locally (1%-2%). CBG-DMH reducing IOP were antagonized by i.p. administration of O-1918 (2.5 mg/kg), a selective antagonist of the abnormal CBD-sensitive cannabinoid-related receptor (CBx), but not influenced by the CB1R antagonist AM251 (2.5 mg/kg) or the CB2R antagonist AM630 (2.5 mg/kg). Contemporary administration of WIN55, 212-2 given at a subthreshold dose to lower IOP (0.25 mg/kg), in association with topical CBG-DMH (0.25%), augmented the IOP-lowering effect of the compound applied alone. These data show that an analog of CBG decreases IOP in normotensive rat eyes independently of CB1R or CB2R involvement, probably activating putative cannabinoid receptors. The greater reduction in IOP seen with co-application of the CB1R agonist WIN55, 212-2 and CBG-DMH, further indicates that effects on the eye induced by CBG-DMH are involving biological targets different from CB1R [79]. Combined, these findings show that CBG may reduce IOP, with this latter study suggesting that synthetic CBG can decrease IOP through mechanisms not involving CB1R or CB2R activation. This finding implies the existence of putative cannabinoid receptors regulating aqueous humor outflow and probably localized to the anterior ocular tissues.

### 3.7. Effects on Skin

The effects of CBG on the expression of keratins 1 and 10, involucrin, and transglutaminase 5, and on DNA methylation of keratin 10 gene, have been studied in the human keratinocytes (HaCaT) cellular line, together with DNA methylation and expression of four DNA methyltransferases (DNMT1, 3a, 3b, and 3L). These experiments showed that CBG caused a significant reduction in the expression of the genes investigated by increasing the DNA methylation of the gene for keratin 10. The data obtained from these experiments led the authors to believe that CBG behaves as a transcriptional suppressor, able to control cell proliferation and cell differentiation, being a substance potentially useful for new therapeutic approaches for skin disorders [80]. On this basis, CBG has been studied as a possible anti-acne drug. Acne is a common skin pathology; however, its more serious expression can deeply weaken the quality of life and, because of social censure, can produce consequent psychological disturbance [81]. The anti-acne effects of CBG on the viability and proliferation of sebocytes were observed through MTT and CyQUAN methods. CBG in a significant way reduced cell viability after a 24 h treatment, but not with treatment lasting 48 h. CBG (10–20 mM) treatment for 24 h significantly reduced lipogenesis stimulated by AEA. These results suggest the possibility that CBG may act as a partial agonist through the same pro-lipogenic signaling pathway on which AEA is active. CBG suppressed inflammation caused by LPS on sebocytes. The results of the above-exposed experiments according to the antiproliferative action of phytocannabinoids, increase the hypothesis that these compounds may be useful in acne and in other skin pathologies associated with inflammatory characteristics, such as psoriasis [82].

### 3.8. Other Effects

It has been suggested that some *C. sativa* derived products might exert positive influence on lower urinary tract symptoms. Similarly, to THC and CBD, CBG is also able to reduce bladder contractility. Experiments with CBG have been conducted on mouse and human isolated bladders. In particular, CBG at concentrations ranging from 10^−8^ to 10^−4^ M, reduced the contractions of mouse bladders induced by acetylcholine, without influencing bladder contractions induced by electrical field stimulation. The results showed that neither rimonabant (10^−6^ M) nor SR144528 (10^−7^M), selective CB1R and CB2R antagonists, modified the inhibitory effect of CBG. CBG also inhibited acetylcholine-induced contractions of human bladders, the effect being significant for the 3 × 10^−5^ M and 10^−4^ M concentrations. These findings indicate that CBG is a more promising compound with respect to other cannabinoids and that it inhibits bladder contraction through a postsynaptic action. The possible involvement of cannabinoid receptors has been hypothesized on the basis of the CBG partial agonism toward CB1R and CB2R and the known effect of CBG on the inhibition of the reuptake of the endocannabinoid anandamide, and finally because components of the endocannabinoid system have a role in the regulation of bladder function. However, the involvement of cannabinoid receptors has not been demonstrated [83, 84].

CBG inhibits platelet aggregation and [^14^C]5-HT release. In a series of experiments, CBG did not inhibit tetradecanoylphorbol acetate (TPA)-induced aggregation of human or rabbit platelets; however, in a dose-dependent way (10^−3^ to 10^−5^ M), it partially inhibited primary aggregation (due to the direct interaction of the aggregating agent with its receptor) and totally inhibited secondary aggregation induced by adrenaline. CBG, dose-dependently also inhibited rabbit and human platelet aggregation stimulated by adenosine diphosphate (IC_50_ of CBG being 2 × 7 × 10^−4^ M) and in rabbit platelets by platelet-activating factor (concentration range to 3 × 10^−5^ M and 10^−4^ M) [85].

CBG and other major cannabinoids such as CBD, CBC, THC, and CBN showed activity *versus* methicillin-resistant *Staphylococcus aureus* (MRSA) strains. The rare occurrence of cross-resistance between microorganisms and plant antibacterial-derived compounds suggests the possibility that CBG could be studied as a potential source of compounds to address antibiotic resistance [86]. CBG also showed activity against *Streptococcus mutans* by altering bacterial membrane properties through the induction hyperpolarization and by reducing membrane fluidity and causing a consequent increase in membrane permeability [87]. Other experiments have demonstrated that CBG is a potential agent against bacterial biofilm, a key factor for contamination of medical devices, favoring the raising of human chronic infections [88].

The appetite-stimulating effects of *C. sativa* have been prevalently assigned to THC, while CBG is also an appetite stimulator. CBG given per os in rats at a dose of 30–240 mg/kg induces an increase in appetite-feeding behavior without any influence on motor activity [89, 90]. Since the loss of muscle, anorexia, and metabolic dysfunction are common consequences of chemotherapy with cytotoxic drugs, *C. sativa* derivatives were used to reduce these effects in oncologic patients. With this aim, the most used were Cannabis extracts containing significant amounts of THC or synthetic THC analogs. Unfortunately, the effect of THC psychotomimetics represents an obstacle to its utilization. For this reason, on the basis of the appetite-stimulating effect of CBG, it has been assessed in laboratory animals whether this cannabinoid given orally for three days reduces anorexia and/or other cachectic signs induced by the i.p. administration of the chemotherapic substance cisplatin (6 mg/kg). CBG was able to reduce the anorexia, weight loss, and metabolic dysfunction caused by cisplatin [91].

**Figure 4:**
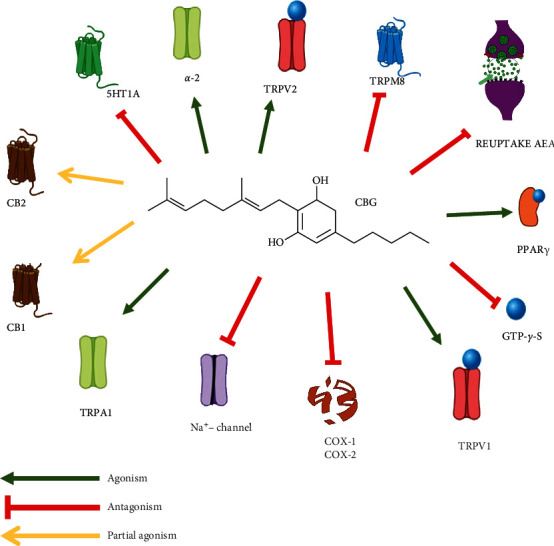
CBG synthetic analogues.

Recently, three new CBG derivatives (HUM-223, HUM-233, and HUM-234) (Figure 4) were synthesized, showing anti-inflammatory and analgesic properties. Additionally, one of them, HUM-234, arrested the development of obesity in mice fed with a high-fat food regimen [92].

## 4. Conclusion

CBG, in its acidic form, is the precursor of the most well-known cannabis derivative compounds, namely, THC and CBD. The study of its pharmacology shows that this compound shares some characteristics with other phytocannabinoids, but it displays its own characteristic profile, as shown by emerging research. Pharmacodynamic research suggests a mechanism of action involving only partial activity of classical cannabinoid receptors, indicating that CBG, as well as CBD, has a multitarget pharmacological action and interacts with a number of endocannabinoid and non-endocannabinoid targets (Figure 3). Research, both *in vivo* and *in vitro*, shows several pharmacological effects of CBG, such as a reduction in IOP and dermatological, anti-inflammatory, antioxidant, antitumoral, and anti-anxiety activities, together with appetite-stimulating effects. Research on CBG also shows the development in the production of synthetic derivatives. Synthetic analogs have been tested with positive effects, particularly in the field of neuroprotection. The promising results obtained with CBG, together with the apparent lack of psychotomimetic THC-like effects, suggest that research on CBG deserves to be deepened as it could be used, alone or in association, for novel therapeutic approaches for several disorders. However, these effects have been proven only through preclinical experiments, and clinical research is needed to confirm these potential activities in humans.

## Abbreviation

ALS: Amyotrophic lateral sclerosis
AEA: Anandamide
ACEA: Arachidonyl-2-chloroethylamide
AUC: Area under the curve
BBB: Blood-brain barrier
CHOP: CCAAT-enhancer-binding protein homologous protein
CBG: Cannabigerol
CBG-DMH: Cannabigerol-dimethyl heptyl
C. sativa: Cannabis sativa
CBGA: Cannabigerolic acid
CBGA-Q: Cannabigerolic acid quinone
CBD: Cannabidiol
CBC: Cannabichromene
CBN: Cannabinol
Δ8-THC: Δ8-tetrahydrocannabinol
Δ9-THC: Δ9-tetrahydrocannabinol
CYP: Cytochrome P
cAMP: Cyclic adenosine monophosphate
CB1R: Cannabinoid receptor 1
CB2R: Cannabinoid receptor 2
CNS: Central nervous system
CRC: Colorectal cancer
CD: Crohn's disease
COX-1: Cyclooxygenase 1
COX-2: Cyclooxygenase 2
MTT: [3-(4, 5-dimethylthiazol-2-yl)-2, 5-diphenyl-2H-tetrazolium bromide)]
DNBS: Dinitrobenzene sulphonic acid
DNMTs: DNA methyltransferases
EAE: Experimental autoimmune encephalomyelitis
GTP*γ*S: Guanosine 5′-O-[gamma-thio]triphosphate
HBMECs: Human brain microvascular endothelial cells
HD: Huntington's disease
H_2_O_2_: Hydrogen peroxide
6-OHDA: 6-hydroxydopamine
5-HT1A: 5-hydroxytryptamine 1A
iNOS: Inducible nitric oxide synthase
IBD: Inflammatory bowel disease
IFN-*γ*: Interferon
IL: Interleukin
i.p.: Intraperitoneal
IOP: Intraocular pressure
LD: Light-dark
LPS: Lipopolysaccharide
LC: Liquid chromatography
GC: Gas chromatography
*C *_max_: Maximum concentration
MAP kinase: Mitogen-activating protein kinase
MS: Multiple sclerosis
MOG: Myelin oligodendrocyte glycoprotein
NSC: Neuroblastoma spinal cord
3NP: 3-nitropropionate
I*κ*B-*α*: NF-kappa-B inhibitor alpha
NF-*κ*B: Nuclear factor-*κ*B
PD: Parkinson's disease
PPAR*γ*: Peroxisome proliferator-activated receptor
QA: Quinolinic acid
ROS: Reactive oxygen species
SOD: Superoxide dismutase
TMEV: Theiler's murine encephalomyelitis virus
*T *_max_: Time to *C*_max_
Tlast: Time of last detection
TNF-*α*: Tumor necrosis factor-*α*
TRP channel: Transient receptor potential channels
TRPA1: TRP ankyrin (A) 1
TRPM8: TRP subfamily melastatin (M) 8
TRPV1: TRP vanilloid (V) 1
TRPV2: TRP vanilloid (V) 2.
